# Artificial Intelligence and Employee Innovation: A Literature Review

**DOI:** 10.3390/bs16101890

**Published:** 2026-10-13

**Authors:** Xi Cun, Liangbo Zhang, Jifan Ren

**Affiliations:** 1 Department of Humanities and Arts, Harbin Institute of Technology, Shenzhen 518055, China; cunxi@hit.edu.cn; 2 Faculty of Business, City University of Macau, Macao 999078, China; 3 School of Economics and Management, Harbin Institute of Technology, Shenzhen 518055, China

**Keywords:** artificial intelligence, individual innovation, employee creativity, human–AI collaboration, innovation management, knowledge management, algorithmic management

## Abstract

Artificial intelligence (AI), particularly generative AI, is increasingly embedded in knowledge work, creative work, customer service, product development, human resource management, and organizational decision systems. Although research shows that AI can raise productivity, support creative output, accelerate searching, and reorganize innovation work, the evidence remains fragmented across innovation management, organizational behavior, information systems, human resource management, economics, human–computer interaction, and behavioral sciences. This framework-based review synthesizes high-quality English-language journal articles on AI, human–AI collaboration, employee creativity, innovative work behavior, algorithmic management, and innovation management. Building on established models of individual innovation, it develops a worker-centered framework linking four innovation stages with three higher-order AI functions: task execution, knowledge support, and organizational governance. The evidence is strongest for searching, idea generation, creativity, and short-term task performance, and remains limited for idea promotion, implementation, diffusion, and realized innovation outcomes. The synthesis identifies three central tensions: performance equalization versus capability differentiation, short-term quality versus long-term capability development, and individual improvement versus collective homogenization. AI can enhance innovation-related work through cognitive expansion, search acceleration, productivity release, recombination, feedback, and learning, but it can also reduce output diversity, weaken expertise development, intensify work, increase dependence, and shift managerial control over creative labor. The review positions AI as both a cognitive resource and an innovation-governance mechanism and outlines directions for future research on mechanisms, heterogeneity, novelty, capability accumulation, and responsible governance.

## 1. Introduction

Artificial intelligence has become a general-purpose organizational technology that supports searching, communication, decision making, design, product development, customer interaction, human resource management, and knowledge coordination. Earlier waves of digital technology primarily automated codifiable and routine tasks, whereas contemporary AI systems can operate in domains that involve language, judgment, images, diagnosis, ideation, classification, and other forms of nonroutine knowledge work. This shift makes AI directly relevant to workers’ individual innovation: the generation, promotion, evaluation, and implementation of novel and useful ideas in organizational contexts.

In this review, employee innovation refers to the individual-level behaviors through which employees identify problems or opportunities, search for knowledge, generate and evaluate ideas, mobilize support, and contribute to implementation. Creativity denotes the production of novel and useful ideas; creative performance concerns the assessed quality of such output; innovative work behavior spans idea generation, promotion, and implementation; and realized innovation refers to the adoption or use of an idea. These outcomes are related but not interchangeable (Amabile, 1988; Anderson et al., 2014; Scott & Bruce, 1994; Shalley et al., 2004; Woodman et al., 1993). We therefore treat productivity and task quality as proximal outcomes rather than direct evidence of full-cycle employee innovation. The review also distinguishes generative AI that produces content, predictive or recommender AI that estimates and ranks options, evaluative AI that scores outputs, and monitoring AI used to allocate, observe, or control work.

The need for a review arises from four forms of fragmentation. First, research on employee creativity and innovative work behavior has developed a rich understanding of motivation, work design, expertise, leadership, climate, affect, and implementation, but much of this work predates the diffusion of generative AI and machine-learning systems into everyday knowledge work. Second, research on AI and innovation management often examines firm-level transformation, product development systems, innovation analytics, or digital innovation capability rather than the worker as the central unit of analysis (Haefner et al., 2021; Mariani et al., 2023; Verganti et al., 2020). Third, information systems and organization theory research has examined algorithms, work, and AI-based decision systems, but these studies are often framed around control, expertise, trust, or occupational change rather than individual innovation (Faraj et al., 2018; Kellogg et al., 2020; Lebovitz et al., 2022). Fourth, recent causal studies of generative AI show productivity and creativity effects, but they do not yet add up to a coherent theory of how AI changes innovation as a staged individual process (Brynjolfsson et al., 2025; Doshi & Hauser, 2024; Jia et al., 2024; Noy & Zhang, 2023; Vaccaro et al., 2024).

For behavioral-science scholarship, this topic is important because AI reshapes how organizational knowledge is accessed, recombined, evaluated, and governed. Rather than functioning merely as tools that accelerate existing tasks, AI systems increasingly operate as knowledge intermediaries, pattern-recognition systems, creative partners, decision aids, monitoring mechanisms, and rule-like infrastructures that influence what workers notice, which ideas they regard as feasible, how they justify novelty, and how organizations allocate attention and resources. Understanding AI-enabled individual innovation therefore requires connecting micro-level creativity and work behavior with knowledge creation, knowledge transfer, innovation capability, and responsible governance.

This review asks three research questions:

- What streams of high-quality research explain how AI affects workers’ individual innovation?
- Through which mechanisms does AI enhance, constrain, or transform individual innovation processes?
- What boundary conditions and future research questions should guide scholarship on AI-enabled individual innovation?

This review makes four contributions. First, it integrates fragmented research around the employee as the focal unit while explicitly distinguishing individual evidence from team- and organization-level boundary conditions. Second, it replaces a flat list of AI roles with a hierarchical taxonomy of task-execution, knowledge-support, and organizational-governance functions across four established stages of individual innovation. Third, it develops a theoretical storyline around three tensions: equalization versus capability differentiation, short-term quality versus long-term capability development, and individual improvement versus collective homogenization. Fourth, it identifies where evidence is strong, where claims remain conceptual, and where future research is needed on idea promotion, implementation, responsible governance, and capability accumulation.

## 2. Research Methodology

### 2.1. Review Design and Scope

This article uses a structured, framework-based review rather than an exhaustive systematic review. Because terminology is dispersed across artificial intelligence, generative AI, machine learning, algorithmic management, human–AI collaboration, employee creativity, innovative work behavior, knowledge work, and the future of work, the purpose is theory-building and integrative synthesis rather than a reproducible census of all publications. The search and screening description below documents the review boundaries and selection logic; it should not be interpreted as full PRISMA compliance.

The focal unit of analysis is the individual employee. The evidence base is separated into three groups: (1) direct empirical studies linking AI-related systems to individual creativity, innovative behavior, learning, task quality, or innovation outcomes; (2) theoretical foundation and review studies used to define constructs and mechanisms; and (3) supplementary frontier studies used to represent emerging phenomena. Team-, organizational-, and system-level articles inform contextual or cross-level boundary conditions but are not treated as direct evidence of individual innovation. The primary publication type is peer-reviewed English-language journal articles in management, economics, innovation, organizational behavior, information systems, human resource management, psychology, and adjacent fields. Outlet quality was used as a secondary quality check, not as a substitute for relevance to the research questions.

### 2.2. Search Strategy

The search was updated on 20 June 2026 using publisher websites, DOI landing pages, web-indexed academic records, and backward and forward snowballing from highly relevant articles. Because no single formal database protocol was retained across the entire search, the review is characterized as framework-based and cannot claim database-level reproducibility. The search terms were categorized into five groups:

- Technology terms: artificial intelligence, AI, generative AI, large language model, machine learning, algorithmic management, algorithmic HRM, human–AI collaboration, automation, augmentation.
- Worker terms: employee, worker, professional, knowledge worker, human capital, work design, job design, HRM, expertise, future of work.
- Innovation terms: creativity, employee creativity, individual innovation, innovative work behavior, idea generation, idea evaluation, new product development, innovation management, creative performance.
- Knowledge terms: knowledge work, knowledge management, knowledge creation, knowledge recombination, absorptive capacity, search, learning, expertise.
- Governance and outcome terms: productivity, learning, diversity, novelty, usefulness, trust, fairness, transparency, control, autonomy, psychological safety, implementation.

Representative Boolean strings included:

- (“artificial intelligence” OR “generative AI” OR “large language model”) AND (“employee creativity” OR “individual innovation” OR “creative performance” OR “innovative work behavior”);
- (“human-AI collaboration” OR “AI augmentation”) AND (“creativity” OR “innovation” OR “worker productivity”);
- (“artificial intelligence” OR “machine learning”) AND (“innovation management” OR “new product development” OR “innovation capability”);
- (“algorithmic management” OR “AI in HRM” OR “algorithmic HRM”) AND (“employees” OR “work design” OR “autonomy” OR “fairness” OR “worker response”);
- (“knowledge work” OR “knowledge management” OR “knowledge creation”) AND (“artificial intelligence” OR “generative AI” OR “algorithmic systems”).

### 2.3. Inclusion and Exclusion Criteria

**Figure 1:**
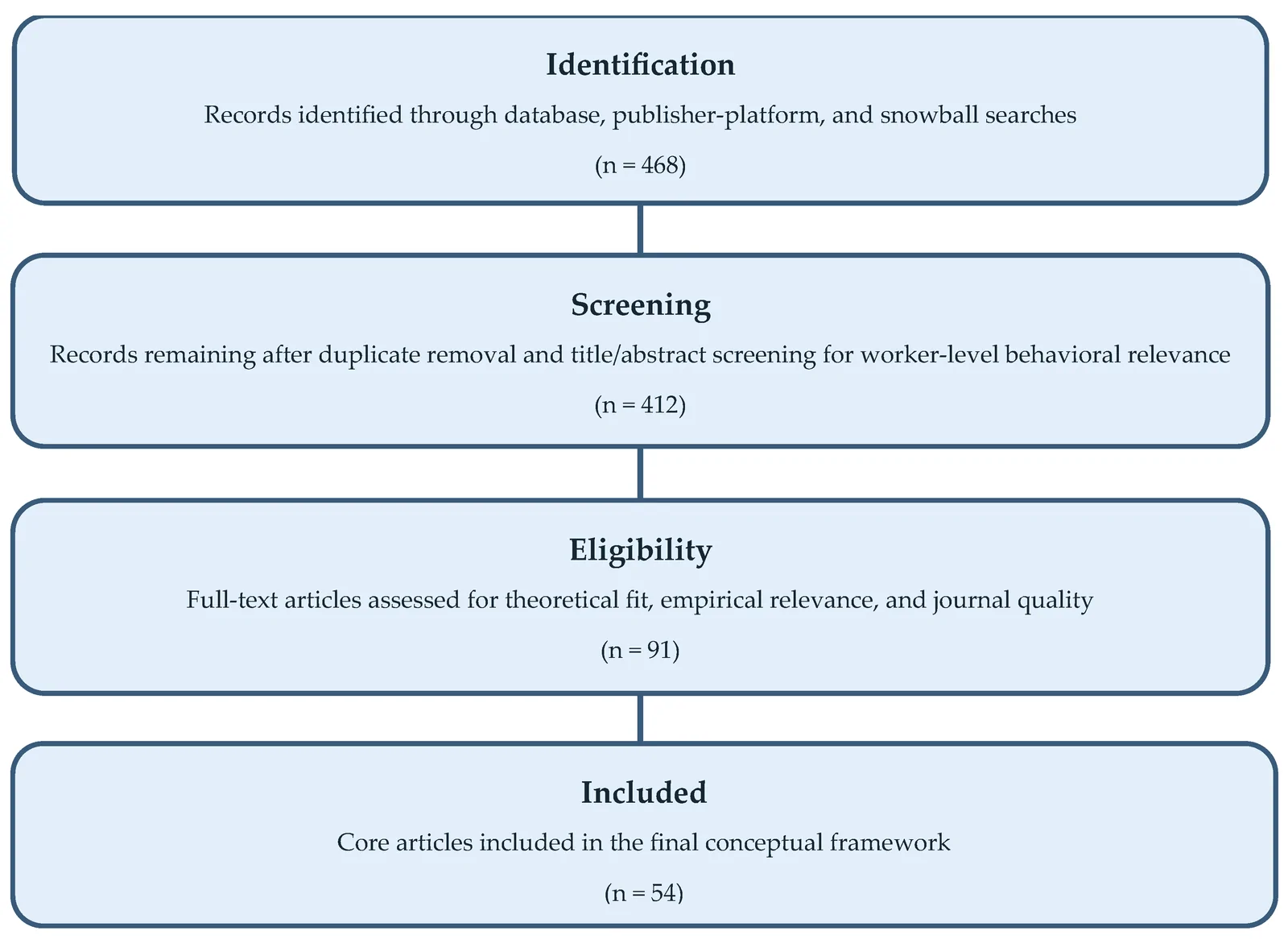
Descriptive summary of the literature selection process.

The search process identified 468 candidate records. After deduplication and relevance screening, 91 full texts were examined, and 54 studies were retained in the expanded corpus table (see Appendix A); adjacent frontier studies were used only as supplementary context. The underlying search log does not preserve a database-by-database yield or a sufficiently granular breakdown of all title/abstract and full-text exclusions. Figure 1 therefore provides a descriptive selection summary rather than a PRISMA flow diagram, and the absence of a fully auditable exclusion log is acknowledged as a limitation.

The selection process is summarized in Figure 1; the reported counts should be read as documentation of this framework-based review, not as evidence of a fully reproducible systematic search.

Studies served one of three explicitly differentiated purposes:

- Direct empirical evidence: studies examining AI, generative AI, machine learning, algorithmic management, or human–AI collaboration in relation to individual creativity, innovative work behavior, learning, task quality, or innovation outcomes.
- Theoretical foundations: high-quality conceptual or review studies used to define creativity, individual innovation, innovation processes, AI functions, knowledge work, algorithmic control, or human–AI trust.
- Supplementary frontier evidence: recent studies used to illustrate emerging mechanisms or contexts without being treated as homogeneous with the direct empirical evidence.

Across all three groups, substantive relevance, methodological clarity, and verifiable bibliographic information were required; journal standing was considered only as an additional quality indicator.

Articles were excluded when AI was mentioned only tangentially, the focus was purely technical without organizational implications, the publication was non-scholarly, the analysis concerned only macro labor substitution without implications for individual work, or bibliographic information could not be verified. Because detailed exclusion reasons were not logged for every record, the corpus should be read as a focused and transparent conceptual synthesis rather than an exhaustive database census.

### 2.4. Coding Procedure

The articles were coded using a theory-informed codebook that was iteratively refined during synthesis. The primary dimensions were: (1) innovation stage—problem framing and knowledge search, idea generation, idea evaluation and selection, and implementation and diffusion; (2) AI function—task execution, knowledge support, and organizational governance; and (3) mechanism—cognitive expansion, productivity release, recombination, feedback, trust calibration, motivation, autonomy, expertise development, output diversity, control, and fairness. Reflexive learning was coded as a feedback process operating across stages, while governance was coded as a cross-stage contextual condition. Additional fields captured theory, standardized method category, empirical context, unit of analysis, AI type, outcome type, key constructs, findings, limitations, and relevance. The author team reviewed the coding during framework development and resolved interpretive differences through discussion; because independent duplicate coding was not completed and a formal intercoder coefficient not obtained, no reliability statistic is claimed.

## 3. Descriptive Overview of the Literature

The reviewed literature falls into seven streams.

First, organizational creativity and individual innovation research provides the conceptual foundation for this review. Classic and contemporary studies distinguish creativity from innovation, identify individual, social, and contextual antecedents, and show that innovative work behavior involves idea generation, idea promotion, and idea implementation (Amabile, 1988; Amabile & Pratt, 2016; Anderson et al., 2014; Janssen, 2000; Perry-Smith & Shalley, 2003; Scott & Bruce, 1994; Shalley et al., 2004; Woodman et al., 1993). This stream highlights intrinsic motivation, creative self-efficacy, autonomy, feedback, leadership, expertise, affect, and climate as important antecedents of individual creative and innovative behavior (Oldham & Cummings, 1996; Tierney & Farmer, 2002; Zhou & George, 2001). However, it has not yet fully theorized AI as a creative collaborator, an evaluative agent, a knowledge intermediary, or a mechanism of workplace control.

Second, AI and innovation management research explains how AI may reshape search, experimentation, new product development, and innovation capabilities. Haefner et al. (2021) argue that AI transforms the organization of innovation by overcoming human information-processing constraints. Mariani et al. (2023) provide a systematic review of AI in innovation research and highlight opportunities around searching, knowledge recombination, and decision support. Gama and Magistretti (2025) classify AI applications in innovation management by capabilities and innovation tasks. Bouschery et al. (2023) focus on transformer-based language models in new product development and propose an AI-augmented double-diamond framework. Kakatkar et al. (2020) and Paschen et al. (2020) further classify AI applications and innovation tasks, while Verganti et al. (2020) argue that AI changes design and innovation by recombining data-driven search with human interpretation. These studies are central to the innovation side of the review, but many remain at the team, process, or firm level.

Third, generative AI productivity and creativity studies provide direct evidence of AI’s effects on individual work. Noy and Zhang (2023) show that ChatGPT 3.5 improves performance on professional writing tasks by increasing speed and output quality. Brynjolfsson et al. (2025) provide field evidence from 5172 customer-support agents and find that a generative AI assistant increased productivity on average, with especially large gains for less experienced and lower-skilled workers. Doshi and Hauser (2024) show that generative AI improves individual creative writing output, especially for less creative writers, while reducing collective diversity because AI-assisted outputs become more similar. Jia et al. (2024) find that AI assistance can augment employee creativity through work-design changes, but the benefits depend on employee skill. Recent field and survey studies further suggest that metacognitive strategies, task complexity, creative process engagement, AI dependence, and sensing capabilities condition the creativity and innovativeness effects of generative AI (Cui et al., 2026; Held & Heubeck, 2025; Sun et al., 2025; Wang et al., 2026).

Fourth, human–AI collaboration and trust research offers boundary conditions. Vaccaro et al. (2024) show in a meta-analysis of 106 experiments that human–AI combinations often improve on humans alone but do not reliably outperform the most competent humans or AI alone. Creative content tasks are more favorable to human–AI gains than finite-choice decision tasks. Glikson and Woolley (2020) review trust in AI and show that trust depends on system tangibility, reliability, task type, transparency, and anthropomorphism. Dietvorst et al. (2015) show that people may avoid algorithms after seeing them err, while Logg et al. (2019) show that people can also display algorithm appreciation. Piller et al. (2024) likewise identify trust as central to generative-AI-enabled innovation. These findings matter because individual innovation involves a mixture of open-ended generation and accountable evaluation, making trust calibration central.

Fifth, information system and organization theory research on AI at work explains how AI reshapes expertise, decision rights, and professional practice. Faraj et al. (2018) conceptualize learning algorithms as technologies that reconfigure work and organizing. Shrestha et al. (2019) theorize how AI changes organizational decision-making structures. Lebovitz et al. (2022) show that professionals may either engage with or resist AI-based recommendations, with important implications for expertise and accountability. Anthony et al. (2023) further explain how occupational knowledge work is reconfigured when technologies mediate professional practice. Together, these studies move this review beyond productivity effects and toward deeper questions about learning, judgment, professional identity, and innovation capability.

Sixth, AI governance, HRM, and algorithmic management research shows that AI does not merely support workers; it can also evaluate, discipline, route, rank, and standardize work. Benbya et al. (2020), Budhwar et al. (2023), Hillebrand et al. (2025), and Vrontis et al. (2022) situate AI within broader organizational, managerial, and HRM systems. Tambe et al. (2019) identify practical and ethical challenges in AI-based HRM, including small data, fairness, accountability, and adverse employee reactions. Kellogg et al. (2020) explain algorithmic control through the mechanisms of directing, evaluating, and disciplining workers. Raisch and Krakowski (2021) theorize the automation–augmentation paradox. Meijerink and Bondarouk (2023), Bankins et al. (2022), and Qin et al. (2023) further show that algorithmic HRM raises concerns about autonomy, value creation, justice, dignity, and perceived fairness. These governance issues are central to individual innovation because innovation depends not only on technical support, but also on autonomy, psychological safety, ownership, fair evaluation, and the perceived legitimacy of decision rules.

Seventh, economic and future-of-work research clarifies AI’s relationship with tasks, skills, and productivity. Autor (2015) argues that technology changes work by altering task content and skill demand rather than simply replacing labor. Acemoglu and Restrepo (2018, 2020) explain how automation and new tasks reshape labor demand and productivity. Felten et al. (2021) propose measures of occupational exposure to AI, while Agrawal et al. (2019) conceptualize AI as lowering the cost of prediction. Although these studies are less directly focused on individual innovation, they help explain how AI may redistribute creative advantage by changing which subtasks become inexpensive, visible, measurable, or strategically valuable.

## 4. An Integrative Framework: AI-Enabled Individual Innovation

The framework employed in this study integrates the literature through a temporal process dimension and a hierarchical AI-function dimension. It is not a claim that every cell has equivalent empirical support. Direct evidence is concentrated in searching, idea generation, creativity, and short-term task performance; idea promotion, implementation, diffusion, and realized innovation remain research gaps.

The temporal dimension draws on established models of creativity and innovative work behavior and comprises four recursively connected stages:

- Problem framing and knowledge searching: recognizing opportunities, diagnosing needs, scanning environments, retrieving knowledge, and forming problem representations.
- Idea generation: producing novel and useful candidate ideas, concepts, explanations, designs, or process improvements.
- Idea evaluation and selection: judging feasibility, usefulness, originality, risk, ethical acceptability, stakeholder fit, and strategic fit.
- Implementation and diffusion: transforming ideas into routines, products, services, processes, or organizational changes.
- Reflexive learning is represented as a feedback loop through which outcomes update problem representations, expertise, and subsequent action; it is not a fifth stage. Organizational governance is a cross-stage condition that shapes access, decision rights, accountability, and the conversion of AI-enabled cognitive resources into innovative behavior. The stages may recur, overlap, or move backward rather than follow a strictly linear sequence.

The second dimension is a hierarchical taxonomy of AI functions:

- Task-execution functions describe the allocation and interaction of work between employee and AI. Automation substitutes for a bounded subtask; augmentation supports a human-controlled task; and collaboration denotes reciprocal, iterative interaction in which employee and system repeatedly shape the evolving output.
- Knowledge-support functions describe how AI retrieves, synthesizes, recombines, represents, and critiques knowledge, provides feedback, and supports learning across stages.
- Organizational-governance functions describe how AI-enabled systems allocate, monitor, evaluate, rank, constrain, explain, and document work. These functions operate across all innovation stages and shape autonomy, fairness, transparency, accountability, and contestability.
- The categories are analytically distinct but may co-occur in one system; for example, a generative model may automate drafting, support knowledge recombination, and be embedded in an evaluative governance process.
- AI type provides an additional boundary condition: generative systems primarily create content, predictive and recommender systems estimate or rank options, evaluative systems score outputs, and monitoring systems observe or direct work. Their effects should not be generalized without regard to function, context, and innovation stage. Table 1 summarizes the hierarchical AI functions across the four stages of employee innovation.

The integration reveals three linked tensions. First, AI can equalize routine output while increasing the premium on advanced framing, judgment, and implementation capabilities. Second, short-term gains in quality and speed can coexist with long-term dependence or skill erosion. Third, higher-quality individual outputs can aggregate into a less diverse organizational idea portfolio. Governance determines whether cognitive resources are converted into innovative behavior by shaping autonomy, voice, verification, ownership, and decision rights. These tensions provide the theoretical storyline for the synthesis below.

## 5. Thematic Synthesis

### 5.1. AI as an Information-Processing and Knowledge-Search Accelerator

Studies in this stream generally converge on the idea that AI expands the volume, speed, and accessibility of information processing. Haefner et al. (2021) argue that AI can overcome human information-processing constraints in innovative work behavior. Mariani et al. (2023) similarly show that AI research in innovation has concentrated on searching, knowledge recombination, decision support, and capability development. Bouschery et al. (2023) propose that transformer-based language models can help innovation teams explore larger problem and solution spaces through summarization, sentiment analysis, insight extraction, and idea generation. These arguments align with the behavioral theory of the firm and knowledge-based views of innovation: innovation depends on searching under bounded rationality, and AI may reduce some search costs by rapidly processing data and generating alternatives.

However, faster searching does not automatically translate into stronger individual innovation. AI may increase the range of options visible to workers, but it may also direct attention toward patterns embedded in training data, organizational repositories, or widely used templates. This creates a tension between search expansion and path dependence. Workers may appear more creative because they generate more alternatives, while the source of variation becomes increasingly concentrated around AI-generated conventions. For this reason, research on AI-enabled innovative work behavior should distinguish among the number of ideas produced, the perceived usefulness of those ideas, their objective novelty, their semantic distance from prior solutions, and their diversity across workers or teams.

The knowledge-management implication is that AI changes the architecture of access. Workers no longer search only through documents, colleagues, routines, or databases; they interact with systems that synthesize and re-present knowledge. This can lower barriers for novices and boundary spanners, but it also creates risks of opacity, hallucination, and invisible standardization. The quality of AI-enabled searching therefore depends on data provenance, prompt diversity, worker expertise, and organizational routines for verification.

**Proposition** **1.** *AI-supported searching increases individual idea novelty when it expands problem representations across distant knowledge domains; reliance on model-default templates weakens this relationship.*

### 5.2. AI and Idea Generation: Productivity, Quality, and Homogenization

The most direct evidence on AI and individual creativity comes from generative AI studies. Noy and Zhang (2023) show that ChatGPT improves performance in professional writing tasks by increasing both speed and quality. Brynjolfsson et al. (2025) find that generative AI support increased customer-support productivity and improved speed and quality, especially for lower-skilled and less experienced agents. Doshi and Hauser (2024) provide an especially important creativity result: access to generative AI improved creative writing output, particularly for less creative writers, but AI-assisted outputs became more similar to one another. Jia et al. (2024) show in an *Academy of Management Journal* study that AI can augment employee creativity by reducing repetitive initial work and allowing employees to focus on higher-level creative problem solving, while also showing that benefits depend on employee skill.

Taken together, these findings suggest that AI can function as a creative equalizer and a creative homogenizer at the same time. It may lift baseline performance by providing workers with draft ideas, structure, language, examples, analogies, and implementation details. Yet because many workers draw on similar model-generated patterns, the collective distribution of outputs may become less diverse. This is critical for organizations: if the goal is to raise the average quality of routine creative work, AI assistance may be valuable; if the goal is radical novelty, differentiation, or breakthrough innovation, unmanaged reliance on the same models and prompts may narrow the search frontier.

The equalizer–homogenizer tension also suggests that creativity should be measured at multiple levels. A single worker’s output may become more fluent and polished, but a unit’s idea portfolio may become more semantically clustered. An organization may therefore experience short-term productivity gains while reducing the option value of diverse experimentation. Future studies should measure not only individual ratings of novelty and usefulness, but also portfolio-level variance, distinctiveness, and downstream implementation success.

**Proposition** **2.** *Generative AI assistance increases the average quality of individual creative output.*

**Proposition** **3.** *Greater similarity in workers’ AI inputs and models reduces the diversity of the collective idea portfolio. The second proposition is explicitly cross-level and requires aggregation measures at the team or organizational level.*

### 5.3. Skill Heterogeneity and the Redistribution of Creative Advantage

A recurring pattern in the evidence is heterogeneity. Brynjolfsson et al. (2025) find that lower-skilled and less experienced workers benefit more from AI assistance than higher-skilled workers, and that AI can help newer agents move more quickly down the experience curve. Doshi and Hauser (2024) similarly find stronger creativity gains among less creative participants. Jia et al. (2024), by contrast, find that higher-skilled employees may be better able to exploit AI assistance for creativity because they can redirect freed resources toward higher-level problem solving. These results are not necessarily contradictory. They suggest that AI may compensate for some capabilities while complementing others.

For individual innovation, the implication is nuanced. AI may democratize access to creative and knowledge resources by helping less experienced workers produce more polished outputs. At the same time, it may change what counts as valuable human expertise. When AI can easily generate drafts, summaries, routine evaluations, or basic code, workers’ distinctive contributions may increasingly depend on higher-order capabilities, such as problem framing, domain judgment, taste, ethical reasoning, experimentation, perspective taking, and implementation. AI may therefore reduce performance gaps in visible routine outputs while increasing the value of deeper and less easily codified innovative capabilities.

This redistribution of creative advantage also has important implications for knowledge management. Inexperienced workers may gain access to expert-like language and routines without necessarily acquiring the tacit knowledge that underlies expert judgment. By contrast, experts may use AI as a cognitive extension that broadens searches, supports recombination, and enables more sophisticated comparison across alternatives. By reducing routine cognitive demands such as drafting, coding, summarizing, or initial information gathering, AI may free cognitive resources for divergent thinking and problem reframing. However, these benefits are unlikely to be evenly distributed. Workers with high creative self-efficacy may use freed cognitive capacity for more ambitious experimentation, whereas workers with lower creative self-efficacy may use AI primarily to produce safer and more conventional outputs. The long-term effect therefore depends on whether AI use is embedded in reflective learning routines or becomes a substitute for deliberate practice.

**Proposition** **4.** *AI assistance reduces skill-related performance gaps in routine creative tasks.*

**Proposition** **5.** *Domain judgment and creative self-efficacy strengthen the effect of AI assistance on high-novelty individual innovation.*

### 5.4. Human–AI Collaboration: From Augmentation to Synergy

Human–AI collaboration research cautions against assuming that combining human and AI capabilities will automatically produce synergy. Vaccaro et al. (2024) show that human–AI combinations often outperform humans alone but do not consistently outperform the most competent standalone human or AI performer. Task type also matters: creative content tasks appear more favorable for human–AI collaboration than finite-choice decision tasks. This distinction is highly relevant to innovation. Idea generation is typically open-ended, iterative, and tolerant of multiple possible answers, whereas idea selection often involves narrower choice sets, ambiguous criteria, accountability, and risk.

AI may therefore be more useful in divergent phases of the innovation process than in convergent phases. Workers can use AI to generate alternatives, identify analogies, reframe problems, and prototype rapidly. At the selection stage, however, AI may increase the risks of over-reliance, misplaced trust, or premature closure. Effective individual innovation may therefore require stage-specific human–AI collaboration routines: AI can be used to expand the space of possibilities, but selection and implementation should still involve verification criteria, domain expertise, stakeholder judgment, and mechanisms for contesting AI-generated recommendations.

Trust calibration is central to this process. Glikson and Woolley (2020) show that trust in AI depends on both system characteristics and task characteristics. Dietvorst et al. (2015) find that people may avoid algorithms after observing their errors, whereas Logg et al. (2019) show that people may also display algorithm appreciation. Both tendencies can undermine innovation. Workers who distrust AI too strongly may miss opportunities to extend or refine promising ideas, while workers who overtrust AI may stop exploring before weaker but potentially valuable ideas have been fully developed.

**Proposition** **6.** *AI has stronger positive effects on individual innovation during divergent generation tasks than during convergent selection tasks, unless workers have clear routines for calibrating trust, verifying outputs, and allocating decision rights.*

### 5.5. AI, Work Design, Motivation, and Creative Agency

Organizational creativity research has long shown that innovation is shaped by motivation, autonomy, resources, support, and work environment. AI can influence each of these conditions. By reducing time spent on routine tasks such as drafting, searching, summarizing, formatting, or documentation, AI may create more room for creative thinking. At the same time, AI may intensify work by raising output expectations, increasing monitoring, accelerating cycles of evaluation and revision, or making workers feel constantly compared against AI-assisted standards. In this sense, a technology that reduces task effort may also introduce new performance pressure.

Consistent with evidence linking AI-enabled job characteristics and substitution concerns to innovative work behavior (Verma & Singh, 2022), the motivational effect of AI depends on whether workers experience it as a resource, assistant, collaborator, replacement threat, or surveillance mechanism. In motivational terms, AI is more likely to support innovative work behavior when it enhances workers’ sense of autonomy and competence, both of which are important for intrinsic motivation. When workers have discretion over how to use AI, they may engage in task crafting by reorganizing parts of their work to create more meaningful and inventive activity. When AI is embedded in rigid workflows or algorithmic evaluation systems, however, workers may comply with prescribed outputs rather than experiment, speak up, or take creative risks. This distinction matters because innovation requires not only idea generation, but also the willingness to deviate from established routines.

Recent evidence on human interaction with generative AI also points to motivational risks: human–generative AI collaboration may improve task performance while undermining intrinsic motivation (Wu et al., 2025). AI may improve task performance while weakening intrinsic engagement if workers perceive reduced ownership, agency, or competence. For innovation, the key issue is whether short-term output gains can be sustained without undermining long-term creative engagement. The most productive configurations are likely to preserve authorship, autonomy, learning, and meaning while reducing unnecessary cognitive burden.

**Proposition** **7.** *Employee-controlled AI use increases innovative work behavior by supporting autonomy and competence.*

**Proposition** **8.** *AI-based surveillance and replacement threat reduce innovative work behavior by weakening creative agency.*

### 5.6. Algorithmic Governance, Fairness, and Creative Voice

AI-enabled individual innovation is not only supportive but also controlling. Tambe et al. (2019) warn that AI in HRM faces challenges related to causal inference, limited data, fairness, accountability, and employee reactions. Algorithmic management research similarly suggests that AI systems can reshape workplace control by directing, evaluating, and disciplining workers (Kellogg et al., 2020). In AI-based HR decision contexts, perceptions of fairness depend on whether employees feel respected, receive understandable explanations, perceive outcomes as acceptable, and regard AI use as legitimate in the relevant decision context (Bankins et al., 2022; Qin et al., 2023).

These issues are directly relevant to innovation. When workers believe that evaluation systems are fair and that mistakes will be treated constructively, they may be more willing to propose novel and uncertain ideas. Organizational justice theory suggests that algorithmic governance shapes both distributive fairness, through AI-mediated evaluation outcomes, and procedural fairness, through the transparency and contestability of AI-supported decisions. These perceptions also affect the psychological contract because workers may infer whether the organization views them as trusted collaborators or as narrowly monitored resources. Algorithmic evaluation can unintentionally punish exploration when systems learn from past success patterns and reward measurable, familiar, or easily codified contributions. As a result, workers may optimize for what is visible to AI-based evaluation rather than pursue more original ideas.

Conversely, well-designed governance can support innovation. Algorithmic systems can make evaluation criteria more transparent, detect overlooked ideas, reduce some forms of human bias in idea screening, and provide feedback that workers can contest or improve. The difference between enabling and suppressing innovation depends on governance design in terms of transparency, contestability, human accountability, privacy boundaries, and recognition of novelty and learning. These design choices influence psychological safety and employees’ willingness to engage in creative voice rather than defensive compliance.

**Proposition** **9.** *Transparent and contestable AI-supported evaluation increases creative voice through procedural fairness.*

**Proposition** **10.** *Opaque or rigid algorithmic criteria reduce innovative work behavior by narrowing the range of acceptable contributions.*

### 5.7. From Short-Term Performance to Long-Term Innovation Capability

Most generative AI studies measure short-term task performance. Individual innovation, however, depends on cumulative capability development. AI may accelerate learning by exposing workers to expert patterns, as suggested by evidence on customer-support agents. It may also reduce opportunities for deliberate practice if workers outsource problem formulation, ideation, or evaluation too early.

This creates a capability paradox. AI can help workers do innovation-related tasks now, but it may either build or erode the human capabilities needed for future innovation. If AI use is reflective, workers may compare alternatives, understand expert reasoning, receive feedback, and improve judgment. If AI use is substitutive, workers may accept outputs without learning why they work. Over time, organizations could become more productive but less capable of generating independent judgment, radical novelty, or domain-specific insight.

Longitudinal research is therefore essential. Future studies should examine whether sustained AI use builds absorptive capacity, creative self-efficacy, domain expertise, and implementation capability, or whether it produces dependence and deskilling. The answer is likely to depend on the design of training, task structures, feedback routines, and organizational expectations. AI may strengthen innovation capability when it is used as a tool for reflection, comparison, and learning, but it may erode capability when it is used primarily as a substitute for problem formulation, evaluation, and deliberate practice.

**Proposition** **11.** *Sustained AI use builds individual innovation capability when it is embedded in reflective learning routines, but erodes capability when it substitutes for problem formulation, evaluation, and deliberate practice.*

## 6. Future Research

### 6.1. Mechanisms: What Exactly Does AI Change in the Innovation Process?

Future research should move beyond aggregated performance effects and examine the mechanisms through which AI changes the innovation process. Does AI improve individual innovation by increasing idea fluency, improving linguistic quality, enabling analogical reasoning, reducing search costs, accelerating feedback, strengthening confidence, or altering motivation? Conversely, does it undermine innovation through anchoring, over-reliance, reduced exploration, deskilling, dependence, or standardized evaluation? Fine-grained process data, prompt logs, version histories, field experiments, and qualitative observations could reveal how workers move between AI-generated suggestions and their own judgment during different stages of innovation.

Research Question 1. Through which cognitive, motivational, behavioral, and social mechanisms does AI influence each stage of individual innovation?

### 6.2. Heterogeneity: Which Workers Benefit, and Which Workers Lose Creative Advantage?

Recent evidence suggests that the effects of AI are unlikely to be uniform across workers with different skills and experience levels. Human capital should be disaggregated into domain expertise, general cognitive ability, digital literacy, AI literacy, creative skills, absorptive capacity, metacognitive strategies, and practical implementation experience. A worker may be strong in writing but weak in domain judgment, or highly imaginative but less effective in implementation. AI may substitute for some capabilities while complementing others. Future behavioral research should also distinguish among task types. Heuristic tasks that depend on imagination, ambiguity tolerance, and judgment may respond differently to AI than algorithmic tasks that follow clearer rules. Cognitive complexity is therefore likely to determine whether AI functions primarily as a resource for exploration or as a mechanism of convergence.

Research Question 2. How do domain expertise, creative self-efficacy, AI literacy, metacognitive strategy, organizational tenure, and task characteristics moderate the effect of AI on individual innovation?

### 6.3. Diversity and Novelty: Does AI Raise the Mean but Shrink the Frontier?

The emerging evidence that AI can increase average creative output while reducing collective diversity raises important questions for innovation research. Organizations need stronger evidence on whether AI-assisted idea portfolios become more similar, whether such similarity affects market differentiation, and how differentiation can be preserved over time. Future studies should therefore examine not only mean performance but also distributional outcomes, including variance, semantic distance from prior art, clustering patterns, extreme-value ideas, and lagged implementation outcomes.

Research Question 3. Under what conditions does AI assistance increase average creative quality while reducing, preserving, or expanding the diversity and frontier novelty of workers’ innovation outputs?

### 6.4. Stage-Specific Design: When Should AI Generate, Critique, or Step Back?

AI can play different roles across problem framing, idea generation, selection, implementation, and learning. Future experiments should manipulate AI roles at specific stages of the innovation process, such as AI as a search assistant, creative collaborator, devil’s advocate, evaluator, implementation assistant, reflective coach, or governance mechanism. Such designs can clarify whether AI should be used continuously or selectively. Rather than treating AI use as a single broad intervention, future research should examine the fit between AI role, innovation stage, task characteristics, and worker capability.

Research Question 4. Which combinations of AI role and innovation stage produce the strongest gains in individual innovation quality, novelty, learning, and implementation?

### 6.5. Governance and Ethics: How Can Organizations Protect Creative Agency?

Future research should integrate AI governance with creativity theory and examine privacy, commercial confidentiality, data provenance, intellectual property, ownership of AI-generated content, bias, hallucination, misinformation, and accountability. Organizations also need evidence on secure model access, data-classification rules, human review, incident reporting, audit trails, vendor controls, and procedures for contesting algorithmic judgments. Theories of psychological safety, procedural justice, work design, and knowledge governance can explain when these arrangements support or suppress innovation.

Research Question 5. How do transparency, contestability, autonomy, ownership rules, and privacy boundaries shape workers’ willingness to use AI for innovative work?

### 6.6. Longitudinal Effects: Does AI Build or Erode Innovation Capability?

Individual innovation depends on cumulative learning. AI may accelerate learning by exposing workers to expert patterns, as field evidence on customer support suggests. Yet it may also reduce opportunities for deliberate practice if workers outsource too much of the creative process. Longitudinal studies should examine whether AI users develop stronger innovation capabilities over time or become dependent on AI-generated scaffolding.

Research Question 6. Does sustained AI use build, redistribute, or erode workers’ innovation capabilities over time, and which learning routines explain these trajectories?

### 6.7. Knowledge and Innovation Outcomes: What Should Organizations Measure?

The final part of the research directions concerns outcomes. Many existing studies rely on productivity, quality ratings, or short creative tasks. Innovation and knowledge scholarship should expand measurement to include knowledge recombination, idea implementation, portfolio diversity, learning transfer, stakeholder adoption, responsible use, and realized value. This is especially important for behavioral-science and innovation scholars because AI may influence not only the production of ideas but also the knowledge processes that determine whether ideas become implemented innovations.

Research Question 7. Which individual-, portfolio-, and organizational-level indicators best capture AI-enabled innovation beyond short-term productivity and output quality?

## 7. Theoretical Contributions

This review contributes to the literature in four ways. First, it consolidates fragmented research in a worker-centered process framework while distinguishing direct evidence from theoretical foundations and supplementary frontier studies. Compared with prior reviews centered on innovation management capabilities or firm-level applications, it makes employee behavior and the uneven evidence across innovation stages explicit.

Second, the review extends creativity and innovation theory by introducing AI as both a cognitive resource and a cognitive infrastructure. Traditional creativity models emphasize individual skills, motivation, work environment, and social context. AI changes each of these elements by altering the availability of ideas, examples, analogies, feedback, templates, evaluation, and coordination.

Third, the review reframes the automation–augmentation debate around individual innovation. AI may automate subtasks, augment workers’ output, collaborate in ideation, and govern behavior simultaneously. The key theoretical challenge is therefore to explain how these roles interact across stages rather than classify AI as either replacement or assistance.

Fourth, the review links micro-level innovation behavior to knowledge governance. AI systems influence not only what individuals can create, but also what knowledge becomes visible, reusable, legitimate, measurable, and accountable. This provides a bridge between employee creativity research and the behavioral-science focus on human cognition, work behavior, and organizational processes.

## 8. Managerial and Policy Implications

For managers, AI should be designed as a portfolio of task-execution, knowledge-support, and governance functions rather than treated merely as a productivity tool. Use can be encouraged for divergent exploration, rapid prototyping, summarization, translation, and feedback, while idea selection and implementation should retain human accountability, domain judgment, stakeholder review, and procedures for contesting AI recommendations.

Managers should also protect diversity. If all workers use the same tools with similar prompts and similar evaluation criteria, creative output may converge. Organizations can reduce this risk by encouraging diverse data sources, multiple models, prompt variation, cross-functional review, independent human ideation before AI exposure, and portfolio-level monitoring of novelty and similarity.

Finally, responsible AI governance should be innovation-sensitive. Organizations should adopt data-classification and approved-tool rules; prohibit disclosure of confidential or personal information to unapproved external systems; define intellectual-property and authorship responsibilities; test for bias and hallucination; require source verification and human sign-off for consequential outputs; maintain proportionate audit trails; and provide incident-reporting and contestability procedures. Monitoring should recognize novelty, learning, experimentation, collaboration, and implementation effort rather than only speed, volume, or surface quality.

## 9. Limitations

This review has several limitations. First, it is a structured, framework-based synthesis rather than a fully reproducible systematic review: a database-by-database search log, complete search strings, record-level exclusion reasons, and independent duplicate coding were not retained, so no PRISMA-compliance or intercoder-reliability claim is made. Second, terminology is evolving rapidly and relevant studies may use different labels. Third, direct evidence is concentrated on generative AI, idea generation, creativity, and short-term task performance; evidence on promotion, implementation, diffusion, realized innovation, and long-term capability remains limited. Fourth, findings from writing experiments, customer-service field studies, surveys, conceptual work, and algorithmic-management contexts are heterogeneous and should not be generalized as if they estimate the same construct or causal effect. Fifth, the English-language journal focus may underrepresent non-English research, industry evidence, and unpublished organizational experiments.

## 10. Conclusions

AI is changing employee innovation by reshaping the conditions under which problems are framed, ideas are generated and evaluated, and proposals are implemented. Current evidence supports cautious conclusions: AI can expand searching, improve average creative output and short-term task quality, and support less experienced workers, but evidence for idea promotion, diffusion, and realized innovation is still limited. The framework shows how task-execution, knowledge-support, and governance functions interact with worker capability, task characteristics, and time. Future research should test whether AI becomes a source of sustained creative augmentation or a mechanism of homogenization, dependence, and control.

## Figures and Tables

**Table 1:** Hierarchical AI functions across four stages of employee innovation.

Innovation Stage/Cross-Stage Process	Task Execution: Automation	Task Execution: Augmentation	Task Execution: Collaboration	Knowledge Support	Organizational Governance
Problem framing and searching	Summarizes documents, mines records, detects patterns	Broadens search scope and surfaces analogies	Helps reframe problems through dialog	Retrieves prior cases and connects distant knowledge (Haefner et al., 2021; Mariani et al., 2023)	Shapes which problems are visible and prioritized (Faraj et al., 2018; Kellogg et al., 2020)
Idea generation	Produces drafts, concepts, variants, code, designs, or prototypes	Increases fluency, flexibility, and elaboration	Supports iterative recombination and counterfactual exploration	Supports recombination, examples, and feedback (Bouschery et al., 2023; Doshi & Hauser, 2024)	May standardize templates and acceptable ideas (Doshi & Hauser, 2024; Kellogg et al., 2020)
Evaluation and selection	Scores, ranks, screens, or clusters options	Provides feedback, risk cues, benchmarks, and simulations	Enables comparison, critique, and red-teaming of alternatives	Provides benchmarks, simulations, and domain heuristics (Glikson & Woolley, 2020; Lebovitz et al., 2022)	Affects transparency, fairness, and contestability (Bankins et al., 2022; Qin et al., 2023)
Implementation and diffusion	Automates documentation, translation, coordination, and routine communication	Speeds up prototyping, stakeholder communication, and troubleshooting	Supports adaptation during rollout	Codifies lessons and implementation knowledge (Bouschery et al., 2023; Verganti et al., 2020)	Tracks performance and accountability (Tambe et al., 2019; Kellogg et al., 2020)
Cross-stage feedback and governance	Produces audit trails and routine reports	Highlights improvement opportunities	Supports after-action reflection	Updates expertise through feedback; may reduce deliberate practice (Brynjolfsson et al., 2025)	Shapes autonomy, voice, ownership, privacy, and psychological safety (Meijerink & Bondarouk, 2023; Bankins & Formosa, 2023)

## Data Availability

The original contributions presented in this study are included in the article. Further inquiries can be directed to the corresponding author.

## Appendix A

**Table A1:** Expanded Core Corpus.

No.	Citation	Journal/Source	Stream	Standardized Method	Outcome Type/Main Relevance
1	Amabile (1988)	*Research in Organizational Behavior*	Creativity foundations	Conceptual study	Creativity/idea generation: Componential theory of creativity
2	Woodman et al. (1993)	*Academy of Management Review*	Creativity foundations	Conceptual study	Creativity/idea generation: Interactionist model of organizational creativity
3	Scott and Bruce (1994)	*Academy of Management Journal*	Individual innovation	Cross-sectional survey	Innovative work behavior: Innovative behavior in generation, promotion, realization
4	Oldham and Cummings (1996)	*Academy of Management Journal*	Employee creativity	Cross-sectional survey	Theory/contextual mechanism: Job complexity and supportive context
5	Zhou and George (2001)	*Academy of Management Journal*	Employee creativity	Cross-sectional survey	Creativity/idea generation: Dissatisfaction and creative response
6	Tierney and Farmer (2002)	*Academy of Management Journal*	Creativity foundations	Cross-sectional survey	Creativity/idea generation: Creative self-efficacy
7	Shalley et al. (2004)	*Journal of Management*	Creativity review	Narrative review	Theory/contextual mechanism: Contextual and personal predictors
8	Janssen (2000)	*Journal of Occupational and Organizational Psychology*	Innovative work behavior	Cross-sectional survey	Innovative work behavior: Innovative behavior under job demands
9	Perry-Smith and Shalley (2003)	*Academy of Management Review*	Creativity/social networks	Conceptual study	Creativity/idea generation: Social network position and creativity
10	Anderson et al. (2014)	*Journal of Management*	Creativity and innovation	Narrative review	Creativity/idea generation: Multilevel creativity and innovation framework
11	Amabile and Pratt (2016)	*Research in Organizational Behavior*	Creativity theory	Conceptual study	Theory/contextual mechanism: Dynamic componential model
12	Autor (2015)	*Journal of Economic Perspectives*	Future of work	Narrative review	Productivity/task quality: Task change and skill demand
13	Acemoglu and Restrepo (2018)	National Bureau of Economic Research	Task execution: automation	Conceptual study	Productivity/task quality: Automation and new tasks
14	Acemoglu and Restrepo (2020)	*Journal of Political Economy*	Task execution: automation	Empirical economics	Productivity/task quality: Robots, tasks, and labor demand
15	Agrawal et al. (2019)	*Journal of Economic Perspectives*	AI economics	Conceptual study	Theory/contextual mechanism: AI for cheaper prediction
16	Felten et al. (2021)	*Strategic Management Journal*	AI exposure	Measurement	Theory/contextual mechanism: Occupational exposure to AI
17	Von Krogh (2018)	*Academy of Management Discoveries*	AI and organization	Essay	Theory/contextual mechanism: Opportunities for AI in organizations
18	Faraj et al. (2018)	*Information and Organization*	Algorithms at work	Conceptual study	Learning/capability: Learning algorithms and organizing
19	Shrestha et al. (2019)	*California Management Review*	AI decisions	Conceptual study	Theory/contextual mechanism: AI and organizational decision structures
20	Raisch and Krakowski (2021)	*Academy of Management Review*	AI management theory	Conceptual study	Theory/contextual mechanism: Automation–augmentation paradox
21	Lebovitz et al. (2022)	*Organization Science*	Expertise and AI	Qualitative study	Theory/contextual mechanism: Professional engagement with AI recommendations
22	Anthony et al. (2023)	*Organization Science*	Technology and expertise	Qualitative study	Theory/contextual mechanism: Occupational knowledge and technological mediation
23	Kellogg et al. (2020)	*Academy of Management Annals*	Algorithmic management	Review/theory	Governance/work experience: Algorithmic control and worker contestation
24	Tambe et al. (2019)	*California Management Review*	AI-HRM	Conceptual study	Governance/work experience: Fairness, accountability, employee reactions
25	Bankins et al. (2022)	*Information Systems Frontiers*	AI-HRM justice	Empirical	Governance/work experience: Dignity and justice perceptions
26	Meijerink and Bondarouk (2023)	*Human Resource Management Review*	Algorithmic HRM	Conceptual/review	Governance/work experience: Autonomy and value creation
27	Qin et al. (2023)	*Journal of Management Information Systems*	AI evaluation fairness	Field experiment	Governance/work experience: Fairness perceptions of AI evaluation
28	Glikson and Woolley (2020)	*Academy of Management Annals*	Trust in AI	Narrative review	Theory/contextual mechanism: Human trust in AI
29	Dietvorst et al. (2015)	*Journal of Experimental Psychology: General*	Algorithm aversion	Experiments	Theory/contextual mechanism: Avoidance after algorithm errors
30	Logg et al. (2019)	*Organizational Behavior and Human Decision Processes*	Algorithm appreciation	Experiments	Theory/contextual mechanism: Reliance on algorithmic advice
31	Haefner et al. (2021)	*Technological Forecasting and Social Change*	AI and innovation management	Review/framework	Theory/contextual mechanism: AI and innovation organization
32	Mariani et al. (2023)	*Technovation*	AI innovation research	Systematic review	Theory/contextual mechanism: AI in innovation research framework
33	Gama and Magistretti (2025)	*Journal of Product Innovation Management*	AI in innovation management	Review/taxonomy	Theory/contextual mechanism: AI applications and innovation capabilities
34	Bouschery et al. (2023)	*Journal of Product Innovation Management*	AI in NPD	Conceptual/catalyst	Theory/contextual mechanism: LLMs and double-diamond innovation
35	Verganti et al. (2020)	*Journal of Product Innovation Management*	AI and design	Conceptual study	Theory/contextual mechanism: Innovation and design in the age of AI
36	Kakatkar et al. (2020)	*Business Horizons*	Innovation analytics	Conceptual study	Theory/contextual mechanism: AI-enabled innovation analytics
37	Paschen et al. (2020)	*Business Horizons*	AI and creativity	Conceptual study	Creativity/idea generation: AI in creative processes
38	Benbya et al. (2020)	*MIS Quarterly Executive*	AI in organizations	Narrative review	Theory/contextual mechanism: AI transformation in organizations
39	Noy and Zhang (2023)	*Science*	Generative AI productivity	Laboratory experiment	Productivity/task quality: ChatGPT and professional writing productivity
40	Doshi and Hauser (2024)	*Science Advances*	Generative AI creativity	Online experiment	Creativity/idea generation: Creativity gains and reduced diversity
41	Brynjolfsson et al. (2025)	*Quarterly Journal of Economics*	Generative AI at work	Field quasi-experiment	Productivity/task quality: Productivity, learning, skill heterogeneity
42	Jia et al. (2024)	*Academy of Management Journal*	AI and employee creativity	Field/empirical	Creativity/idea generation: AI assistance and employee creativity
43	Vaccaro et al. (2024)	*Nature Human Behaviour*	Human–AI collaboration	Systematic review and meta-analysis	Productivity/task quality: Collaboration effects and task boundaries
44	Piller et al. (2024)	*Journal of Applied Behavioral Science*	GenAI and innovation trust	Conceptual/empirical	Theory/contextual mechanism: Trust in AI-generated innovation outputs
45	Sun et al. (2025)	*Journal of Applied Psychology*	GenAI and creativity	Field experiment	Creativity/idea generation: Metacognitive strategies and employee creativity
46	Wang et al. (2026)	*European Journal of Innovation Management*	GenAI and creativity	Cross-sectional survey	Creativity/idea generation: Creative self-efficacy and process engagement
47	Held and Heubeck (2025)	*Digital Business*	GenAI and innovative work behavior	Cross-sectional survey	Theory/contextual mechanism: Sensing capability and GenAI evaluation capability
48	Wu et al. (2025)	*Scientific Reports*	Human–GenAI collaboration	Laboratory experiment	Productivity/task quality: Performance and intrinsic motivation tradeoff
49	Cui et al. (2026)	*International Journal of Information Management*	AI dependence	Empirical	Creativity/idea generation: Dual effects of AI dependence on creativity
50	Verma and Singh (2022)	*Computers in Human Behavior*	AI job characteristics	Cross-sectional survey	Innovative work behavior: AI-enabled job characteristics and innovative work behavior
51	Vrontis et al. (2022)	*International Journal of Human Resource Management*	AI in HRM	Systematic review	Theory/contextual mechanism: AI-HRM literature and future research
52	Budhwar et al. (2023)	*Human Resource Management Journal*	AI and people management	Critical review	Theory/contextual mechanism: Ethical lens on AI people management
53	Bankins and Formosa (2023)	*Journal of Business Ethics*	AI ethics and meaningful work	Conceptual study	Theory/contextual mechanism: AI deployment, meaningful work, autonomy, and ethical implications
54	Hillebrand et al. (2025)	*Academy of Management Annals*	AI management	Integrative review	Theory/contextual mechanism: Human–AI collaboration and algorithmic management
